# Recapitulating the Angiogenic Switch in a Hydrogel-Based 3D In Vitro Tumor-Stroma Model

**DOI:** 10.3390/bioengineering8110186

**Published:** 2021-11-15

**Authors:** Claudia Kuehlbach, Sabine Hensler, Margareta M. Mueller

**Affiliations:** Molecular Cell Biology Laboratory, Institute of Technical Medicine, Hochschule Furtwangen University, 78054 Villingen-Schwenningen, Germany; claudia.kuehlbach.17@ucl.ac.uk (C.K.); sabine.hensler@gmx.de (S.H.)

**Keywords:** angiogenesis, tubular structures, vascular sprouting, high-throughput 3D tumor-stroma model, tumor–stroma interaction, dextran hydrogel

## Abstract

To ensure nutrient and oxygen supply, tumors beyond a size of 1–2 mm^3^ need a connection to the vascular system. Thus, tumor cells modify physiological tissue homeostasis by secreting inflammatory and angiogenic cytokines. This leads to the activation of the tumor microenvironment and the turning of the angiogenic switch, resulting in tumor vascularization and growth. To inhibit tumor growth by developing efficient anti-angiogenic therapies, an in depth understanding of the molecular mechanism initiating angiogenesis is essential. Yet so far, predominantly 2D cell cultures or animal models have been used to clarify the interactions within the tumor stroma, resulting in poor transferability of the data obtained to the in vivo situation. Consequently, there is an abundant need for complex, humanized, 3D models in vitro. We established a dextran-hydrogel-based 3D organotypic in vitro model containing microtumor spheroids, macrophages, neutrophils, fibroblasts and endothelial cells, allowing for the analysis of tumor–stroma interactions in a controlled and modifiable environment. During the cultivation period of 21 days, the microtumor spheroids in the model grew in size and endothelial cells formed elongated tubular structures resembling capillary vessels, that appeared to extend towards the tumor spheroids. The tubular structures exhibited complex bifurcations and expanded without adding external angiogenic factors such as VEGF to the culture. To allow high-throughput screening of therapeutic candidates, the 3D cell culture model was successfully miniaturized to a 96-well format, while still maintaining the same level of tumor spheroid growth and vascular sprouting. The quantification of VEGF in the conditioned medium of these cultures showed a continuous increase during the cultivation period, suggesting the contribution of endogenous VEGF to the induction of the angiogenic switch and vascular sprouting. Thus, this model is highly suitable as a testing platform for novel anticancer therapeutics targeting the tumor as well as the vascular compartment.

## 1. Introduction

Tumor development, growth and progression is not solely mediated by tumor cells but requires a growth- and angiogenesis-promoting tumor stroma. The stroma or microenvironment contains cellular components such as fibroblasts, immune and inflammatory cells, blood vessels and components of the extracellular matrix (ECM). This plays a crucial role not only in physiological tissue homeostasis but also in tumorigenesis and progression [1].

Solid tumors need a connection to the vascular system to ensure nutrient and oxygen supply for growth beyond a size of 1–2 mm^3^ [1,2,3,4]. To achieve this, tumor cells alter physiological tissue homeostasis, e.g., through the secretion of inflammatory cytokines and angiogenic factors and activate the tumor microenvironment [1,2,3]. They mediate vessel formation by inducing the so called “angiogenic switch”, an alteration in the balance of pro- and anti-angiogenic factors in the tumor microenvironment to the proangiogenic side [1,5]. This results in the transition from an avascular tumor to an increased tumor vascularization [6]. While there are many angiogenic factors identified so far, VEGF (Vascular Endothelial Growth Factor) is still considered one of the main factors that induce vascular sprouting [2,6,7]. VEGF has several different subtypes, i.e., VEGF-A, VEGF-C and VEGF-D, that each bind to different VEGF receptors (VEGFR). This leads to specific functions., e.g., VEGF-A binds to VEGFR-2 on blood vessel endothelial cells and promotes blood vessel angiogenesis [2,6]. VEGF-C and –D bind with high affinity to the VEGFR-3, that is, e.g., expressed on lymph endothelial cells and thus contribute to the induction lymphangiogenesis [6]. In addition to VEGF, basic fibroblast growth factor (bFGF) as well as platelet-derived growth factor (PDGF) are also positive regulators of angiogenesis [3,6,7], that contribute to the proliferation of vessel-associated cells such as pericytes. Expression of proangiogenic factors is correlated with environmental stress, such as hypoxia, nutrient deprivation, acidosis and the formation of reactive oxygen species (ROS), but also with the deregulation between oncogenes and tumor suppressor genes [3,6]. While physiological angiogenesis as observed in embryonal development, wound healing and the female cycle is clearly mediated by endothelial cells and vessel associated cells, tumor angiogenesis is not exclusively restricted to endothelial cells [8]. Tumor cells themselves are able to form new vessels [9,10,11] in a process called vascular mimicry. During vascular mimicry, tumor cells form a network of tubular structures which are perfusable and rich in matrix proteins [12,13,14], and start to express endothelial- as well as tumor-cell markers. While these tumor-cell-derived vessels are significantly less functional compared to endothelial-cell-lined vascular structures [15,16], their formation clearly decreases the dependence of tumor growth on angiogenesis and allows the escape from antiangiogenic treatments which in turn can be associated with poor prognosis [4,6].

Consequently, a better understanding of the interplay between angiogenesis vascular mimicry and tumor therapy is required for successful cancer treatment. To clarify these complex interactions, so far predominantly 2D cell cultures and animal models have been used. However, these mostly lead to poor transferability of the experimental results to the in vivo situation in patients [17,18,19,20,21]. In this context, 3D cell culture technology is of particular importance. Compared to traditional 2D cell cultures, 3D in vitro cell culture models reflect much better the situation in the in vivo tissue context. They also allow a well-controllable manipulation in a humanized system, whereas animal experiments suffer from significant physiological difference to the human organism [22]. As an equivalent of the extracellular matrix, tissue extracts such as rat collagen or Matrigel are most frequently used in these 3D models due to their biocompatibility and biological activities [23,24]. For these matrices, there are also first models that allow the combination of tumor cells with different stromal cell types in 3D [1,25,26,27,28,29] to analyze the critical contribution of tumor–stroma interactions to growth and progression of tumors. However, the use of biological tissue extracts as extracellular matrix equivalents may suffer from uncontrollable variations in batch-to-batch composition resulting in poor reproducibility of the data. This makes such models unsuitable for use in standardized efficacy tests in the pharmaceutical industry. Therefore, despite their limited informative value, 2D is often preferred to 3D cell culture for these standardized tests.

Here, the importance of biomimetic hydrogels of synthetic origin comes into play. Biomimetic hydrogels have a chemically defined, controllable and reproducible composition, and thus allow the establishment of 3D cell culture models as well-defined systems with finely tunable modifications [22,29]. Hydrogels consist mainly of two components, the polymers and the crosslinkers, which are linked via reactive groups such as thiol, maleimide and amino groups to form an elastic network. However, unlike the ECM molecules of the natural matrix, these synthetic polymers lack the multifunctional binding sites for the cells’ integrins that activate signaling cascades essential for cell survival, function and structure. Natural ECM molecules additionally allow cell-mediated matrix modification via cleavage sites for specific proteases. To enable a physiological interaction of cells with the hydrogel matrix, adhesion ligands, e.g., RGD peptides as well as protease (e.g., MMP) cleavage sites must additionally be coupled or included into the polymers of the synthetic matrix. Examples of such synthetic hydrogels are PEO- (polyethylene oxide), PEG- (polyethylene glycol), PVA- (polyvinyl alcohol) and dextran-based hydrogels that can be combined with various biodegradable (containing protease cleavage site) and nondegradable crosslinkers. PEO-based hydrogels have already been approved by the FDA as carrier materials for tissue engineering [30] and PEG is frequently used in copolymers as scaffold for drug delivery and tissue engineering [31].

Dextran polymers such as the 3-D Life hydrogels used in this study provide significant advantages compared to PEO or PEG gels, as they allow the enzymatical degradation of the hydrogels by dextranase. This facilitates the release of the cells embedded in the hydrogels and thus permits further analyses, e.g., by gene expression or blotting techniques. The MMP cleavage sites that are incorporated in the crosslinker of the 3-D Life hydrogel allow the generation of matrices that are cleaved by cell-derived MMPs. This can map important cell–ECM interactions in tumorigenesis, progression, and metastasis. The cell–matrix interactions relevant for efficiently mimicking natural ECM can be selectively controlled in the 3-D Life hydrogels by covalent immobilization of peptides (e.g., RGD) on polymer components or by the controlled addition of individual ECM components to the polymerization solution. As such, the dextran-based hydrogel has proven invaluable in the establishment of a highly standardized 3D model to analyze –stroma interactions in a human cell context. This 3D tumor-stroma model encompasses tumor cells, stromal fibroblasts and inflammatory cells, and allows us to determine tumor cell contribution to the activation of the surrounding microenvironment [29]. While this model reflects a large part of the tumor–stroma interactions, it lacks the vessel compartment in the tumor tissue and thus prohibits the targeting of the angiogenic process by specific drug candidates in a 3D in vitro model.

Testing of antiangiogenic drug candidates requires the detailed analysis of the different mechanisms that contribute to the induction of tumor angiogenesis. Here, the spheroid model as the equivalent of a microtumor is increasingly used, as it is able to capture the complex processes during the activation of the “angiogenic switch” [21]. Due to insufficient diffusion of oxygen and nutrients, spheroids such as microtumors acquire a necrotic core where induction of a hypoxia response and thus of proangiogenic mediators can be observed [3,32,33]. As such, tumor spheroids are a useful tool for the research on progression from “dormant” microtumors to a vascularized tumor entity. The combination of tumor cells and endothelial cells in multicellular spheroids shows that the endothelial cells form tubule-like structures within the spheroid [32,34,35,36]. At the same time, there are reports stating that fibroblasts within the tumor stroma are essential for supporting the endothelial cells in their invasion into the cancer cell aggregates [37,38,39,40]. To comprehensively reflect the interactions of the tumor microenvironment in vivo, inflammatory cells should also be integrated into the 3D tumor models in addition to tumor cells, endothelial cells and fibroblasts [33,41], e.g., tumor-associated macrophages are known to promote angiogenesis by secreting proangiogenic factors to activate endothelial cells [33,41,42], while neutrophils (differentiated to a tumor-supporting phenotype) are essential for angiogenesis, as they secrete MMP-9 into the tumor microenvironment. This results in degradation of the ECM and the release of matrix-bound growth factors, such as VEGF [41,43,44].

Thus, in order to bring a 3D, in vitro, tumor-stroma model as close as possible to the in vivo situation, endothelial cells, inflammatory cells and stromal fibroblastsneed to be included in addition to tumor spheroids [45,46]. Further, to make the model suitable for standardized testing of anticancer drugs in the pharmaceutical industry, the use of a synthetic hydrogel as matrix is necessary. This type of model allows the investigation of early stages in the interaction between tumor spheroids and endothelial cells [47], in an in vivo-like, tumor-stroma context, giving insights on the tissue changes associated with the angiogenic switch and the promotion of tumor angiogenesis. Therefore, we modified the standardized 3D tumor tissue model described before [29] and added endothelial cells and tumor spheroids into the hydrogel. Using this novel modification, it is possible to analyze in detail the interaction between microtumor spheroids and endothelial cells, as well as the inflammatory compartment and fibroblasts in the tumor stroma in an in vivo-like setting.

## 2. Materials and Methods

### 2.1. Cell Lines

Fibroblasts: Human dermal fibroblasts and red fluorescent protein (RFP) transfected fibroblasts (Pelobiotech, Martinsried, Germany) were grown in Dulbecco’s modified Eagle’s medium (DMEM), 10% fetal calf serum (FCS) and 1% penicillin/streptomycin (10,000 U/10,000 µg/mL Biochrom, Berlin, Germany) (D10), and subcultured at a split ratio of 1:3 upon confluency.

Immune cells: U937 cells and HL60 cells were maintained in RPMI 1640 Medium (Life Technology, Darmstadt, Germany) with 10% FCS and 1% penicillin/streptomycin (10,000 U/10,000 μg/mL) (RPMI 10), and subcultured twice a week at a cell concentration of 1 × 10^6^ cells/10 mL medium.

Two days prior to establishing the 3D model, the differentiation of U937 cells into macrophages was induced by a 48 h incubation with 50 nM phorbol 12-myristate-13-acetate (Sigma-Aldrich, Steinheim, Germany) in RPMI 10, as described [48].

For differentiation of HL60 cells into neutrophils, cells were incubated for four days at a density of 3 × 10^6^ cells per 75 cm^2^ flask in 30 mL RPMI 10 containing 1.25% DMSO, as described [49]. Medium was changed once after 2 days. Success of differentiation was determined based on cell morphology of the adherently growing cells. Nonadherent cells were removed by washing with phosphate-buffered saline (PBS).

Tumor cells: H838 and H838-GFP: The human non-small-cell lung carcinoma cell line H838, and H838 cells transfected with pTracer CMV2, containing the coding sequence for the green fluorescent protein GFP (Fisher Scientific, Schwerte, Germany), were maintained in D10 medium containing 200 µg/mL zeocin or in D10 medium (H838 untransfected) and were subcultured twice a week.

GFP-transfected HCT 116 colon carcinoma cells (HCT116 GFP) were maintained in MEMα medium containing 10% FCS, 1% penicillin/streptomycin (10,000 U/10,000 µg/mL) and 200 µg/mL G418 and were subcultured twice a week.

Microtumor spheroids were generated three days prior to the preparation of the hydrogel cultures. The spheroids, containing 20 cells in a total volume of 20 µL of medium, were initialized using the hanging drop method under the lid of a 10 cm culture dish containing 10 mL of PBS. Spheroids were incubated under standard conditions for three days.

On day three, the spheroid droplets were harvested into a 1.5 mL tube. To reduce the volume, the spheroids were centrifuged at 100 rpm for 2 min. The supernatant was fully removed, and the spheroids were carefully resuspended in the appropriate amount of PBS (see Table 1).

Endothelial cells: Human umbilical vein endothelial cells (HUVEC) and GFP transfected HUVEC (Pelobiotech, Martinsried, Germany) were maintained in Endothelial Cell Growth Medium (MVEM, Pelobiotech, Martinsried, Germany) containing all supplements and subcultured at 80% confluency.

### 2.2. Preparation of Hydrogel

3-D Life Dextran-CD Hydrogel cultures (Cellendes GmbH, Reutlingen, Germany) were prepared in 24-well insert plates (Corning, Wiesbaden, Germany) as described before [29] in a total volume of 150 µL. Components 1–3 (Table 1) were mixed, RGD peptide was added and the mix was incubated for 20 min (min) at room temperature. Finally, CD-Link as well as cells and spheroids in PBS (Table 1) were added. The gel was transferred into a 24-well transwell insert plate (Corning, Wiesbaden, Germany) and left to polymerize for 1 h at 37 °C and 5% CO_2_. Subsequently, MVEM without the VEGF supplement was added and changed again after 1 h. For one of the experiments, VEGF supplement was added to the endothelial cell growth medium.

For the 96-well format, the total volume of the hydrogels was reduced to 50 µL and transferred into a 96-well transwell insert plate (Corning, Wiesbaden, Germany). Cell numbers were reduced accordingly (Table 2). For better identification of the single cells within the hydrogel, red fluorescent protein (RFP)-transfected fibroblasts were used (Pelobiotech, Martinsried, Germany). For further analysis, conditioned medium was retrieved on day 7, 9, 11, 14, 16, 18, and 21.

Evaluation: The hydrogels were cultivated over a period of 21 days. Medium was changed three times a week. The changes in cell morphology and proliferation were documented by microscopic photography. For treatment with cisplatin (Biotechne, Wiesbaden, Germany), two different concentrations were added to the culture medium. Treatment started at day 7 of the hydrogel cultures. Number of fibroblasts was counted with Photoshop C7S and Spheroid size was measured using Image J.

### 2.3. VEGF-ELISA

Conditioned media of the hydrogel cultures were analyzed with respect to the concentration of VEGF in the medium using the Human VEGF Quantikine ELISA Kit (R&D Systems, Wiesbaden, Germany) according to the manufacturer’s instructions. Experiments were performed in duplicates.

## 3. Results

### 3.1. Formation of Tubular Structures in the 3D Tumor-Stroma Model

The dextran-hydrogel-based 3D tumor-stroma model presented here mimics the complex cell–cell and cell–matrix interactions of the in vivo context. By integrating different stromal cell types into a hydrogel containing microtumor spheroids, it provides a model for cell communication between tumor and stromal cells reflecting their essential role in tumor growth and progression [50,51,52]. Thus, it creates an optimal environment for standardized testing of the impact of different therapeutic substance candidates on tumor and stroma.

The experimental setup in this study includes not only cells of the innate immune system, such as macrophages and neutrophils and connective tissue fibroblasts, but also endothelial cells to allow the analysis of potential drug candidates targeting the vascular compartment.

Over a period of 20 days, growth and differentiation of the stromal cell types and the H838 tumor spheroids in the hydrogel cultures was documented microscopically. Within this observation time, elongated tubule-like structures appeared in the vicinity of the tumor spheroids. Special attention was given to the morphology of the tubular structures, such as their bifurcation, complexity and general expansion as well as to cells that had attached to these structures. In general, an origin or center from which structures extended could be identified for each tubular structure.

**Figure 1:**
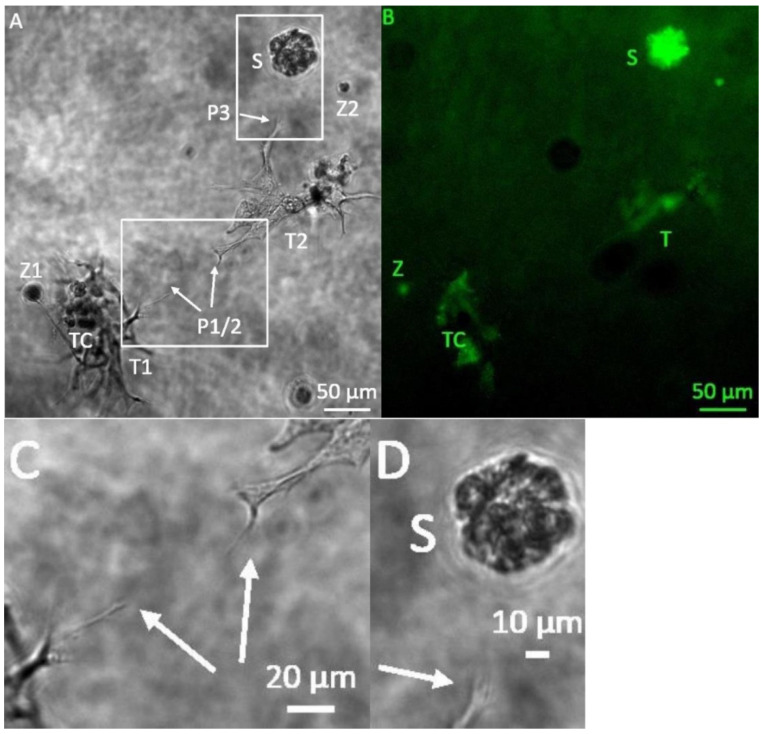
Representative microphotographs of 3D hydrogel tumor-stroma cultures on day 6. (**A**): Tubule-like structures showed increased bifurcation after a cultivation period of 6 days. From the tubular structure T2 in the middle, one structure moves upwards (arrow P3) towards the H838 tumor spheroid (S). One bifurcation of each tubular structure T moves towards each other (arrows P1/2). The left structure (T1) also shows strong cell attachments (TC). In addition, a spherical cell accumulation (Z1) and a small roundish cell accumulation (Z2) is visible. (**B**): GFP fluorescent micrograph of A. The spheroid (S) is strongly GFP-positive, as are the cell attachments (TC) and the spherical cell accumulations (Z1/2). (**C**): Enlarged section at P1/2. (**D**): Magnified section at P3.

Starting six days after the initiation of the 3D tumor-stroma cultures, tubule-like structures appeared, forming fine bifurcations. In some cases, structures with bifurcations originating from different centers spread out towards each other (Figure 1C). Furthermore, from day 6 on, individual bifurcations of the tubule-like structures extended in the direction of spheroids (Figure 1D). Accumulation of roundish structures containing GFP-positive cells that differed in their appearance from the typical tumor spheroid was observed (Figure 1B). Microphotographs taken in phase contrast and fluorescent channel suggest that these were H838-GFP tumor cells associated with the tubule-like structures (Figure 1A,B).

**Figure 2:**
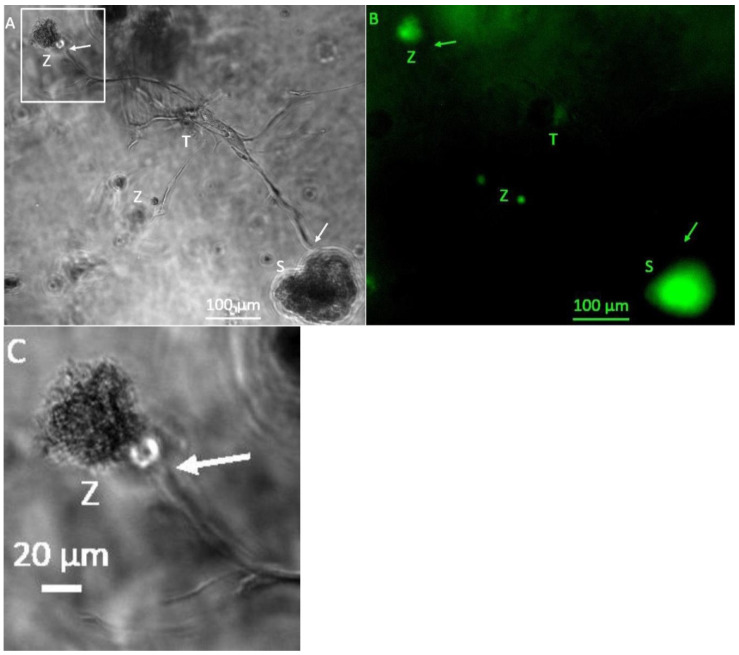
Representative photomicrographs of a 3D hydrogel tumor-stroma culture on day 13 (**A**): central tubular structure (T), individual cells and cell clusters (Z) and a tumor cell spheroid (S). Bifurcation of the tubular structure and growth into the direction of the H838 tumor spheroid, or the cell accumulation in the upper left corner of the picture (arrows). The spheroid shows a roundish shape, which appears slightly dented below the bifurcations extending towards it. (**B**): GFP fluorescent image of (**A**). The individual cells and cell accumulation (Z) and the spheroid are GFP-positive. (**C**): Magnified section of (**A**), marked in (**A**).

From day 13 on, connecting tubule-like structures could be observed between tumor-cell spheroids (Figure 2A). GFP-positive (Figure 2B), round cell clusters that, unlike singular tumor-cell spheroids, exhibited a rough surface with small appendages, were observed (Figure 2A,C). The tubule-like structures that had formed on day 13 (Figure 2A) had already reached a maximum size beyond which no further growth could be observed on the following days. Addition of VEGF supplement to the MVEM medium used did not alter size or bifurcation of the tubule-like structures.

**Figure 3:**
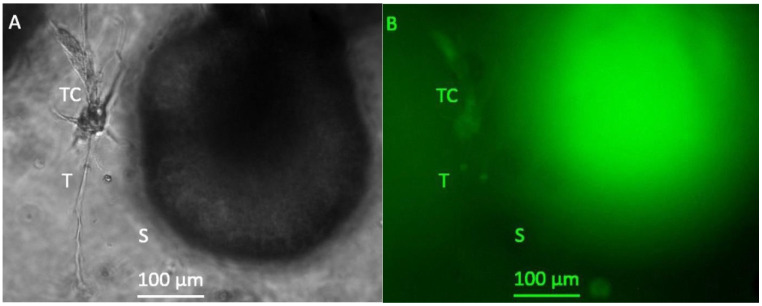
Representative photomicrographs of a 3D hydrogel tumor-stroma culture on day 20 (**A**): A tubular extension (T) with cell attachments at the origin (TC) is located in direct vicinity to a very large spheroid (s) (**B**): GFP fluorescent picture of (**A**). The spheroid is completely GFP-positive, the cell attachments (TC) at the tubular structure are also slightly GFP-positive. The fine tubular extension (T) is GFP-negative.

After a cultivation period of 20 days, spheroid growth had further increased and spheroids with a diameter of several hundred μm and a very high density could be observed (Figure 3). However, no further growth of tubule like structures beyond those observed on day 13 could be detected.

### 3.2. Miniaturization for High-Throughput Screenings

To better accommodate the needs of high-throughput testing for pharmaceutical substances, the hydrogel-based 3D tumor-stroma model was miniaturized from a 24-well to a 96-well format using a total volume of 50 µL. To allow the reduction in volume while still maintaining an adequate number of spheroids (17) with an initial volume of 20 µL each, excess media had to be removed from the spheroid suspension before adding it to the culture. This could be achieved either by sedimentation, here 60 min proved the optimal time interval, or by centrifugation. Different centrifugation times and speeds were tested and a centrifugation of 2 min at 100 rpm proved most effective in reducing the media volume sufficiently and leaving the tumor spheroids intact for integration into the 3D culture. 

Initially, spheroids and cell-containing hydrogels were pipetted into standard 96-well plates and cultivated over a period of 21 days. Medium was changed three times a week. Already after 10 days, cell number in the gels decreased drastically, which is an effect that was especially obvious towards the bottom of the hydrogel cultures. This suggested that diffusion of oxygen and nutrients from the media on top of the hydrogel culture to the bottom of the well was not sufficient. To ensure sufficient nutrient and oxygen supply of the 3D hydrogel cultures, cultivation was adapted to 96-well transwell culture plates (Corning, Wiesbaden, Germany). In these insert plates, the hydrogel-based 3D tumor-stroma models could be successfully cultivated for a period of 21 days.

**Figure 4:**
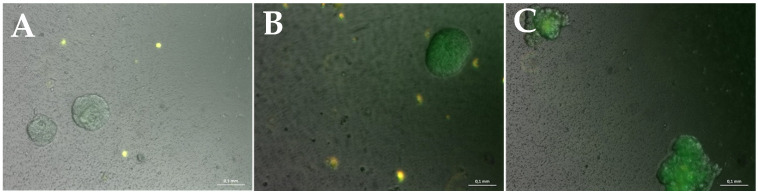
Merged brightfield, GFP and DsRed fluorescent pictures of a presentative hydrogel culture. GFP-positive microtumor-spheroids can be observed over the entire cultivation time, and grow in size. Additionally, RFP-positive fibroblasts exhibiting a roundish shape can be detected. (**A**): After 7 days of cultivation. (**B**): After 14 days of cultivation. (**C**): After a cultivation time of 21 days. Measuring bar: 100 µm.

While GFP-positive H838 tumor spheroids grew consistently in these 50 µL cultures, in 96-well plates, no tubule-like structures could observed (Figure 4).

Inclusion of RFP-positive fibroblasts in the cultures revealed their survival during the entire observation period, however the RFP fibroblasts did not exhibit the physiological spindle-shaped morphology that was previously observed for these stromal fibroblasts in the 3D tumor model (Figure 6).

The lack of tubular structures in these smaller volume cultures suggested a disbalance in the very important communication between the individual cell types in the 3D tumor-stroma model. Therefore, different cell and spheroid numbers were integrated into the 50 µL hydrogel cultures to achieve an optimized communication via soluble mediators of angiogenesis and cell activation. The optimal cell numbers that were determined are listed in Table 3.

**Figure 5:**
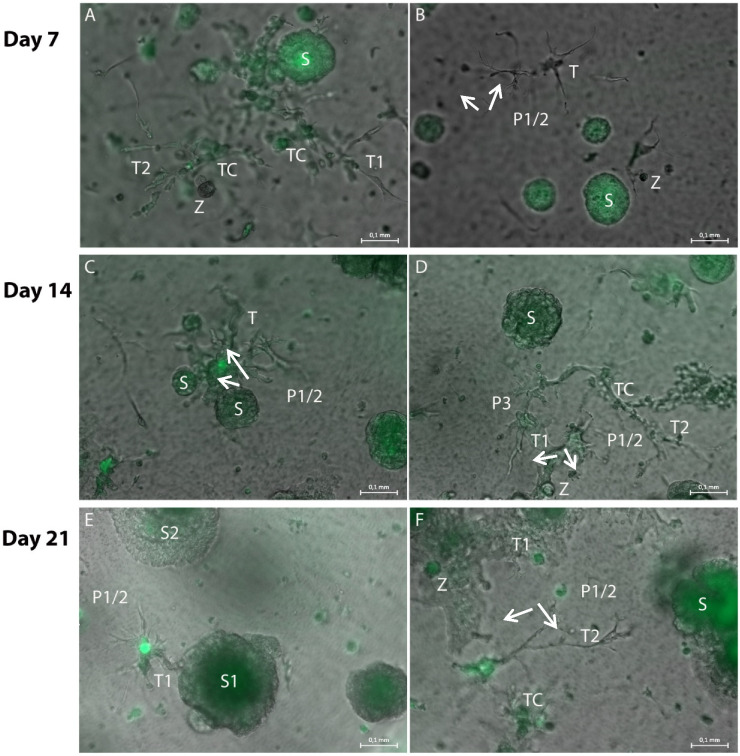
Representative merged brightfield and GFP fluorescence photomicrographs of hydrogel cultures on day 7 (**A**,**B**), 14 (**C**,**D**) and 21 (**E**,**F**) in a 96-transwell plate. (**A**): Tubule-like structures (T1 and T2) with GFP-positive cells attached (TC) are located in close proximity to a spheroid (S). The fine tubule-like extensions are GFP-negative. (**B**): The end tips (P1/2 arrows) of the tubule-like structure (T) extend towards the spheroid (S). They are GFP-negative, as are the spherical cell attachments (Z). (**C**): Wider tubule-like structures (T) extend both towards and from the spheroid (P1/2 arrows, S). (**D**): The wide tubule-like structures (T1 and T2) are GFP-negative, as are the fine bifurcations (P1/2 arrows, P3), but they exhibit GFP-positive cell attachments (TC). There are also spherical cell attachments (Z) visible. (**E**): The GFP-negative tubule-like structures (T) show more extensions as well as bifurcations (P1/2 arrows). They mainly extend from the spheroid (S1) and towards a second spheroid (S2). (**F**): The tubule-like structures (T1) build a dense GFP-negative network which includes smaller GFP-positive spherical cell attachments (Z). Fine tubular-like extensions (P1/2 arrows) extend towards a bigger tumor-cell spheroid (S). Measuring bar: 100 µm.

The hydrogel cultures containing these numbers of cells could be cultured over a period of 21 days (Figure 5). Over the whole cultivation period, tubule-like structures could be observed which grew in width over the entire time. The tubule-like structures were always GFP-negative and were located in close proximity to GFP-positive spheroids. They sometimes had GFP-positive cell attachments either as single cells or spherical cell aggregates (Figure 5C,F). With longer cultivation time (more than 7 days), tubule-like structures extended into branched networks which could include small, GFP-positive cell aggregates (Figure 5C,D).

**Figure 6:**
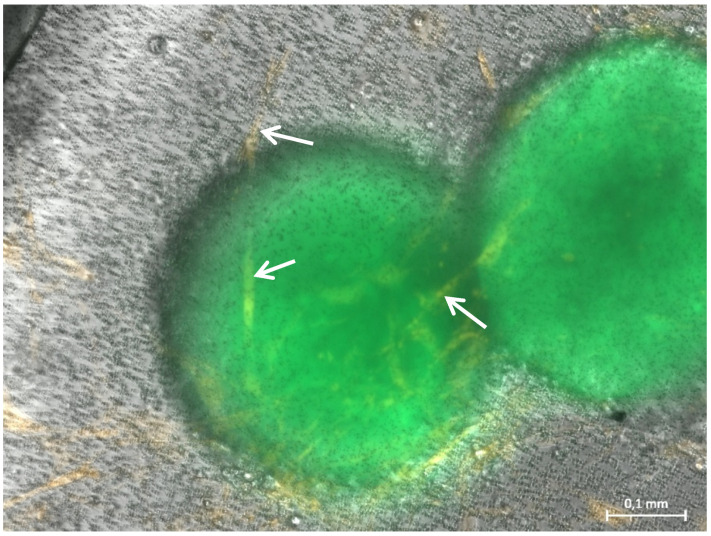
Representative merged brightfield and GFP/RFP fluorescence picture of a hydrogel culture on day 11, containing spheroids of GFP-transfected HCT116 (green), RFP-transfected fibroblasts (orange), HUVEC cells, macrophages and neutrophils. In these cultures with optimized cell numbers, fibroblasts show their spindle-shaped morphology (arrows). Measuring bar: 100 µm.

Spindle-shaped fibroblasts grown in 3D cultures are known to form a network of single cells with thick cell bodies and fine extensions [53]. In the cultures containing the optimal number of cells to assure an efficient cell–cell communication, RFP fibroblasts showed again their typical spindle-shaped morphology and connected to networks, as was previously observed in the 24-well format hydrogel cultures (Figure 6).

To ensure that the tubule-like structures seen in the dextran-hydrogel-based 3D tumor-stroma models are indeed formed by endothelial cells and not, e.g., by fibroblasts, GFP-transfected HUVEC cells (Pelobiotech, Martinsried, Germany) and untransfected H838 tumor-cell spheroids were included in the cultures.

**Figure 7:**
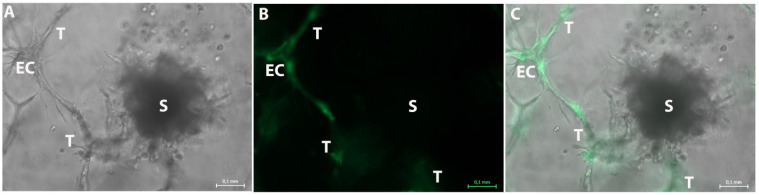
Representative photographs of the 3D tumor-stroma model with GFP transfected HUVEC and tumor spheroids of untransfected H838 tumor cells on day 20. (**A**): Brightfield picture shows a spheroid (S) and an endothelial cell network (EC). The ECs form several tubule-like structures (T) in all directions. (**B**): GFP fluorescence photo of A also shows the GFP-positive endothelial cell (EC) within the tubule-like structures (T). The spheroid (S) is GFP-negative. (**C**): Merged picture of (**A**,**B**). The GFP-negative spheroid (S) and the GFP-positive endothelial cell (EC) with the tubule-like structures (T) can be observed. These structures extend into all directions. Measuring bar: 100 µm.

Figure 7 confirms that GFP-positive HUVEC cells form the tubule-like structures. They extend into all directions and also connect to the tumor-cell spheroids. These results clearly confirm that the tubule-like structures that could be observed in the hydrogel-based 3D tumor-stroma models are formed by endothelial cells, and may represent a first step in vessel sprouting during the angiogenic switch.

Taken together, the dextran-hydrogel-based, 3D, in vitro tumor-stroma model established in this work excellently reflects the in vivo tumor microenvironment. By including microtumor spheroids, stromal fibroblasts, inflammatory cells (macrophages and neutrophils) and endothelial cells, the system allows an in vivo-like cell interaction in the tumor microenvironment. Without the addition of external growth factors or mediators of tumor-stroma activation, cell–cell interaction in the tumor microenvironment of these cultures initiates the sprouting of tubule-like structures from endothelial cells, thereby reflecting the first step of the angiogenic switch in a tumor tissue.

### 3.3. VEGF-ELISA

VEGF is considered the main angiogenic factor to induce vascular sprouting [3,6,7]. In the tumor microenvironment, the tumor supports vessel development by secreting different cytokines such as bFGF and VEGF, to recruit endothelial cells into the vicinity of the tumor and to induce the formation of a tumor supporting vasculature [3,6,7]. To determine whether an enhanced secretion of VEGF in the 3D tumor-stroma cultures could be responsible for the formation of the observed tubule-like structures, VEGF was measured in the conditioned media by ELISA.

**Figure 8:**
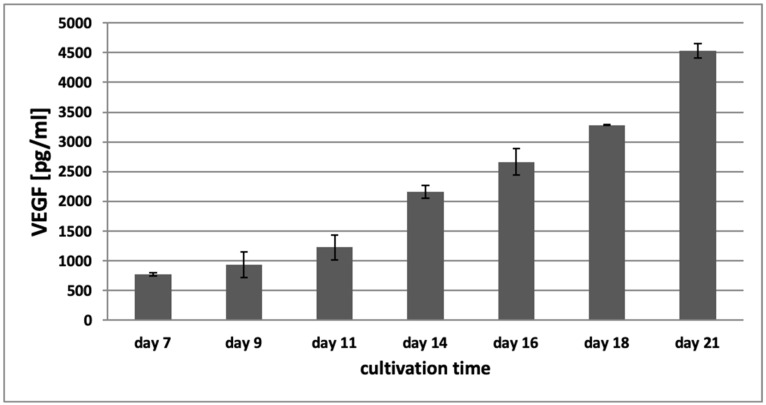
VEGF concentration in conditioned media of 3D hydrogel-based tumor-stroma cultures as determined by ELISA. The concentration of VEGF increases continuously over the course of 21 days in tumor-stroma models containing H838 spheroids, reaching a level almost six-fold above the initial VEGF concentration at day 21. Experiments were performed in duplicate.

Figure 8 shows the concentration of VEGF in conditioned media of the 3D hydrogel tumor-stroma cultures on day 7, 9, 11, 14, 16, 18, and 21. On day 7, the concentration of VEGF in the conditioned medium of the hydrogel cultures was 774.5 pg/mL (±29.23 pg/mL). It continuously increased over the cultivation time and reached a concentration of 4528.5 pg/mL (±117.14 pg/mL) on day 21, suggesting that this increase in VEGF might contribute to the induction of the angiogenic switch and the sprouting of tubular structures that can be observed in the 3D tumor-stroma cultures.

### 3.4. Validation of the Model by Treatment with Cisplatin

**Figure 9:**
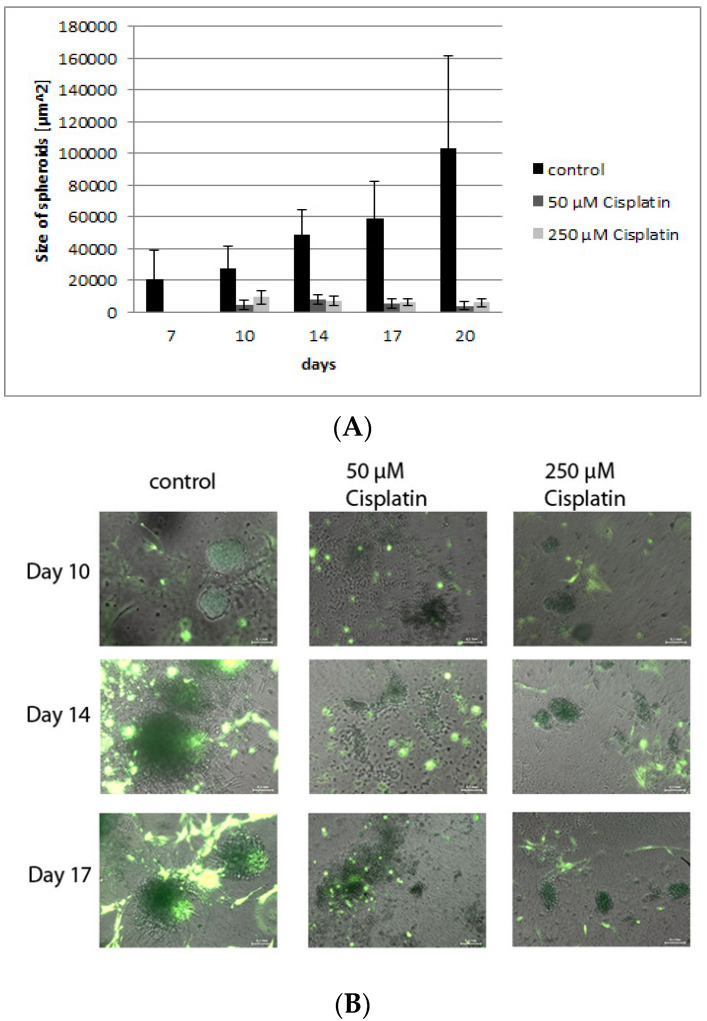
(**A**): The bar graph shows a significant reduction in spheroid size after treatment with both concentrations of cisplatin compared to control. (**B**): Fluorescence photomicrographs of hydrogel cultures containing H838GFP tumor spheroids (dark green), RFP fibroblasts (light green), inflammatory cells and endothelial cells after 10, 14 and 17 days of treatment with 50 μM and 250 μM. In comparison to control cultures, cisplatin does not reduce the number of fibroblasts but negatively affects the fibroblast network as well as fibroblast morphology which changes towards small spindle shaped cells. Measuring bar: 100 µm.

Cisplatin is known to be a very potent and frequently used chemotherapeutics in various cancers that generates its effects by inducing DNA lesions [54,55]. The 3-D Life hydrogel cultures presented here were treated with two different concentrations of cisplatin to determine the efficacy of chemotherapeutic treatment in the culture model, and to compare the effects to those seen in vivo [54,55]. Figure 9A shows photomicrographs of tumor-stroma hydrogel cultures treated with different concentrations of cisplatin after 10, 14 and 17 days. While the number of stromal cells is unaffected by the treatment, the network of fibroblasts is reduced and their morphology has changed towards small spindle-shaped cells. The sizes of the tumor spheroids of H838GFP cells are significantly decreased by cisplatin treatment (Figure 9A,B) confirming an effect of the drug on proliferating tumor cells comparable to the in vivo data in cancer treatment studies [54], while leaving stromal cells predominantly unaffected.

## 4. Discussion

Tumor-induced angiogenesis is essential for tumor formation and is characterized by abnormal and permeable blood vessels and deregulated function of endothelial cells [56,57,58,59,60]. The inhibition of angiogenesis leads to an explicit inhibition of tumor growth and is therefore a promising approach for anticancer therapies [51,56]. To develop effective antiangiogenic drugs, an in-depth understanding of the entire cascade underlying the initiation of angiogenesis is essential [22,61,62].

While tumor cells themselves play a crucial role in inducing an angiogenic response [1,2,3,4,5,6], e.g., by recruiting endothelial cells into the tumor vicinity by the secretion of cytokines, such as bFGF and VEGF [2,6,7], there is also a critical role played by additional cells in the tumor microenvironment. Inflammatory cells such as neutrophil granulocytes and macrophages contribute to angiogenesis and tumor progression in vivo by differentiating to a tumor-supporting phenotype [52,63,64]. Fibroblasts support the development of a protumor, proangiogenic environment, e.g., through the secretion of platelet-derived growth factor (PDGF), fibroblasts growth factor (FGF), epithelial growth factor (EGF) and also hepatic growth factor (HGF), by extracellular matrix proteins, and fibroblast activation protein (FAP) [65,66]. Thus, successful tumor vascularization is the result of the fine-tuned interaction between numerous cell types in the tumor stroma that needs to be reflected in a 3D in vitro model in order to analyze the efficacy of antiangiogenic and antitumor therapies.

As a consequence, the use of 2D models in the prognosis of pharmaceutical efficacy and toxicity for anticancer drugs deals with error rates as high as 95% [67] due to their lack of in vivo like cell–cell interactions and 3D tissue organization [68,69]. While animal or xenograft models are certainly much better-suited in providing a 3D tissue context, the significant physiological differences make results from animal model often not transferable to humans. This frequently leads to an abortion of clinical trials for hopeful anticancer drug candidates that were successfully tested in animal models [70,71,72]. Therefore, 3D in vitro cell culture models play increasingly important roles in preclinical substance tests [73].

In this work, we introduce a 3D tumor-stroma model that recapitulates the angiogenic switch in a controlled in vitro environment without the external addition of proangiogenic factors. The model is based on a bioinert dextran hydrogel as ECM equivalent. While other matrices such as rat collagen I or Matrigel recapitulate the physiological ECM and also allow the combination of tumor and stromal cells in an 3D culture system, they have an uncontrollable batch-to-batch variability that leads to poor reproducibility and prohibits standardization [1,22,27,29]. This makes models using either collagen I or Matrigel as ECM replacement unsuitable for standardized efficacy tests of anticancer drugs as required in the pharmaceutical industry. In contrast, synthetic hydrogels can be modified by adding crosslinker and adhesion sites to satisfy specific system requirements [74,75], thereby providing an in vivo-like ECM while still maintaining a chemically defined and standardized matrix environment.

The inclusion of stromal cells such as fibroblasts, inflammatory cells and endothelial cells together with microtumors in a 3D system, as shown in this study, authentically reproduces the in vivo situation in the tumor microenvironment. As previously shown, the model reflects the induction of a tumor-supporting inflammatory compartment, as determined by the differentiation of macrophages and neutrophils included in the system into a tumor supporting M2 and N2 phenotype, respectively [28,29]. It also allows the analysis of the communication between endothelial cells, the remaining stromal cells and tumor microspheroids at a very early stage, namely, when the angiogenic switch is turned [47]. Thus, it gives the opportunity to test the impact of antiangiogenic therapies alone or in combination with other therapeutic approaches in a well-controllable, 3D, in vitro situation [45,46].

In accordance with an in vivo-like cell–cell interaction, in the hydrogel-based 3D tumor-stroma model presented here, tubular structures are formed by the HUVEC endothelial cells without any addition of external cytokines to the cultures. The tubular structures often grow in close proximity or seem to extend towards the tumor spheroids or other areas of high cell density. In agreement with the increase in tumor spheroid volume and the resulting increase in the necrotic core of the spheroids, the VEGF concentration in the conditioned media of the culture increases continuously over the whole cultivation time, suggesting that the VGF produced by tumor and stromal cells in these cultures could be responsible for the induction of endothelial cell sprouting. To test the hypothesis that VEGF secreted by tumor cells is sufficient for endothelial cell sprouting in the model, 3D cultures cultivated with MVEM, with and without added VEGF, were compared. There was no significant difference between cultures with and without added VEGF in the medium. The size and growth of the spheroids was almost identical, and the formation of tubular structures was also comparable in number, size and lumen in both approaches. This confirms that the endogenous VEGF that is secreted by the tumor and stromal cells in the systems is sufficient to induce the formation of tubular structures by endothelial cells.

To date, there are a number of reports in the literature that describe the formation of tubule-like structures by HUVEC in the presence of tumor cells. However, all reports so far require the external addition of growth factors to the system to achieve the formation of these structures or report the use of VEGF-containing matrices such as Matrigel., e.g., HUVEC cells are known to form tubule-like structure networks on Matrigel when grown in conditioned medium of M2 macrophages [76]. In collagen gels and also in Matrigel based cultures, angiogenesis can be initiated, however this succeeds only when the tumor growth factor TGF- β is added to the culture [77]. Chen et al. could show that HUVEC-coated dextran beads which were suspended in a fibrin gel as a natural matrix and cultured in an endothelial cell growth medium (EGM-2) containing VEGF as supplement form tubule-like structures when cultivated with either human glioma cells or by adding additional VEGF [78]. In 2015, Bray et al. were able to show that the formation of tumors and vascular-like structures occurs in cultures containing tumor cells mesenchymal stromal cells, and HUVEC endothelial cells in a modified soft (star- PEG) hydrogel and in Matrigel, when supplemented by addition of 5 μg/mL of each VEGF, FGF-2 and SDF 1 [22]. Investigations from Roudsari and colleges on lung adenocarcinoma cancer cells (344SQ) with endothelial cells and pericytes encapsulated in cell-adhesive, proteolytically degradable poly(ethylene) glycol-based hydrogels, show that 344SQ formed spheroids in hydrogels and secreted proangiogenic growth factors that significantly increased with exposure to transforming growth factor beta 1 (TGF-β1), a potent tumor progression-promoting factor. To study tumor cell–vascular cell interactions, this group cultured a 2-layered PDMS hydrogel slide containing 344SQ and HUVEC cells in EGM-2 medium supplemented with VEGF. They observed an enhanced formation of invasive clusters at the interface between the tumor cell and the endothelial cell containing hydrogel [79]. So far, only Correa de Sampaio have demonstrated a coculture system of endothelial cells, fibroblasts and tumor cells that exhibited presprout formation of endothelial cells in EGM-2 media, even in the absence of exogenously added VEGF. However, this system relies on the use of collagen as a natural matrix, and collagen is known to bind VEGF from the EGM-2 media that was used to establish the endothelial cell spheroids with high affinity, suggesting that the system still contained significant amounts of VEGF [39].

In the hydrogel-based 3D tumor-stroma model presented here, the formation of tubular structures by HUVEC endothelial cells can for the first time be demonstrated in an artificial matrix without the capacity of binding residual VEGF as well as in culture media without VEGF or any other external growth factor as supplement. The tubular structures often grow in close proximity or extend towards the tumor spheroids or other areas of high cell density. This might be due to the fact that in areas with high density of activated fibroblasts, inflammatory cells or tumor cells, a higher amount of chemoattractant and angiogenic factors should be present. In agreement with this, Kalluri and Zeisberg, 2006, observed growth and elongation of tubular structures towards areas of higher cell density in hydrogel 3D cell cultures [65].

It is well known that necrosis and hypoxia in the center of spheroids lead to the secretion of VEGF, bFGF und TGF-β, which in turn activate endothelial cells and induce angiogenesis [4,80,81,82,83,84]. Similarly, neutrophil granulocytes in the tumor microenvironment secrete VEGF and MMP-9, thereby enhancing the development of new vessels [64,85,86,87]. Neutrophil-secreted VEGF affects endothelial cells by inducing their proliferation and migration, the latter being enhanced by the MMP-9-induced matrix degradation. At the same time VEGF promotes the secretion of IL-8 which in turn recruits more neutrophils to the area generating a positive feedback loop that ultimately results in sustained angiogenesis [85,88]. Similarly, a high number of tumor-associated macrophages lead to an increased production of angiogenic factors such as VEGF, PDGF, IL-8, TNF-α und bFGF [85,89,90,91] and an enhanced expression of angiogenesis promoting enzymes such as MMP-2, MMP-7, MMP-9 and MMP-12 [85,92,93]. These metalloproteases then degrade the ECM allowing endothelial cells to migrate and at the same time enable the release of cytokines such as VEGF from the degraded ECM [85]. In agreement with these studies, we hypothesize that the N2 neutrophils and M2 macrophages that were shown to be present in these cultures [28,29] contribute significantly to the observed vascular sprouting.

Some of the tubular structures that can be observed appear to have a lumen, similar to the study described by Sukmana and Vermette in 2010, albeit without exogenous growth factor addition that was required there [94]. Whether this lumen consists exclusively of endothelial cells, cannot be distinguished at this point, as a lot of GFP-positive cells are attached to the tubular structure. In tumor angiogenesis, it is known that neovascularization sometimes occurs as a mosaic, with tumor cells forming vessel structures together with endothelial cells [3,10,95]. Indeed, there is significant evidence that the formation of new vessels within the tumor microenvironment is not restricted to endothelial cells [8] but can also be established by tumor cells [10,11]. This process of tumor cell-mediated vessel formation is called vascular mimicry and affords the tumor some extent of independence from angiogenesis [4]. Yang et al. could demonstrate that vascular mimicry is induced by tumor-associated fibroblasts. In a 3D in vitro model of hepatocellular carcinoma, the formation of tubular structures was absent as long as there were no fibroblasts in the cultures. After addition of cancer-associated fibroblasts to the culture, the development of vascular mimicry structures could be observed and was dependent on fibroblast-derived TGF β and SDF-1 [96]. During the development of vessels through vascular mimicry, tumor cells dedifferentiate and adopt an endothelial-cell-like phenotype [97,98], leading to vessels that are lined by tumor cells instead of endothelial cells. In later stages of tumor growth, the vessels formed by vascular mimicry merge with endothelial-cell-derived vasculature to form mosaic hybrid structures, as was shown in a breast cancer model where vascular mimicry was induced by decreased levels of VEGF and lack of inflammatory stimuli [98,99]. Measurement of VEGF in the conditioned media of the 3D tumor-stroma cultures confirmed a significant increase in VEGF concentration during the observation period. This increase of VEGF over time could be associated with the increased angiogenic activity within the hydrogel, and may recapitulate observations in tumor tissues in vivo, where an increased level of VEGF is seen in many patients with different cancers, and can be correlated with advanced tumor progression and a poor prognosis [100,101,102].

Together with the abundant presence of activated tumor-supporting inflammatory cells, the measured increase in VEGF suggests that the tubular structures observed in the hydrogel-based 3D tumor stroma cultures indeed originate from endothelial cells. This was further confirmed by introducing GFP-transfected HUVEC cells into the 3D in vitro tumor-stroma model. The results showed that the tubular structure seen in the cultures are lined exclusively by endothelial cells and therefore can be understood as the initialization of angiogenesis in the hydrogel-based 3D tumor-stroma model described here.

## 5. Conclusions

To better mimic the in vivo situation in a tumor, including the induction of angiogenesis, the hydrogel-based 3D tumor-stroma model established by Hensler et al. [29] was extended to include endothelial cells and microspheroids of tumor cells. Comparable to in vivo, the microspheroids show an increase in size over the cultivation period of three weeks. At the same time, the endothelial cells introduced into the model sprouted to form tubular structures even without the addition of exogenous growth factors, such as VEGF. These tubular structures extend in close proximity to or towards the tumor spheroids, and even connect with each other. Consequently, the model presented here is highly suitable as a testing platform not only for anticancer therapies but also for antiangiogenic drug candidates for the pharmaceutical industry.

## Figures and Tables

**Table 1:** Components and volumes for the preparation of the hydrogel cultures.

Components	Reagents	Volume/µL for 150 µL Hydrogel (24-Well Format)	Volume/µL for 50 µL Hydrogel (96-Well Format)
1	10× CB, pH 7.2	10	3.5
2	Water	53.5	18
3	SG-Dextran (28 mmol/L SH-reactive groups)	15	5
4	RGD-Peptide (20 mmol/L SH groups)	4	1.4
5	CD-Link (20 mmol/L SH groups)	17.25	5.75
6	Cells in PBS	50	17

**Table 2:** Cells used for the preparation of the 3D tumor-stroma model. Total volume 150 or 50 µL.

Cells	Cell Number per 150 µL Hydrogel	Cell Number per 50 µL Hydrogel
U937, differentiated to macrophages	6522	2200
HL-60, differentiated to neutrophils	5000	1700
Fibroblasts	5000	1700
HUVEC	5000	1700
H838 GFP microspheroids	50 spheroids	17 spheroids

**Table 3:** Optimal cell numbers to integrate into the miniaturized hydrogel in a total volume of 50 µL.

Cells	Cell Number per 50 µL Hydrogel
U937, differentiated to macrophages	2500
HL-60, differentiated to neutrophils	2000
Fibroblasts	2000
HUVEC	2000
H838 GFP microspheroids	21 spheroids

## Data Availability

The data presented in this study are available in the present article “Recapitulating the Angiogenic Switch in a Hydrogel-Based 3D In Vitro Tumor-Stroma Model”.
